# Human Papillomavirus Vaccine Discourse and Sentiment on Reddit Before and After COVID-19: Mixed Methods Retrospective Cross-Sectional Study

**DOI:** 10.2196/83558

**Published:** 2026-05-19

**Authors:** Marcin Marciniak, Sean A Setzen, Deeya Bhattacharya, Jamie Masliah, Robin Powszok, Erich M Sturgis, Vanessa C Stubbs, Mihir K Bhayani

**Affiliations:** 1 Department of Otorhinolaryngology-Head and Neck Surgery, Rush University Medical Center, 1611 W Harrison St Suite 550, Chicago, IL, 60612, United States, 1 (312) 942-6100; 2 Rosalind Franklin University of Science and Medicine, North Chicago, IL, United States; 3 Department of Otorhinolaryngology-Head and Neck Surgery, St. Luke’s University Health Network, Bethlehem, PA, United States; 4 Rush University Medical College, Chicago, IL, United States; 5 Department of Otolaryngology-Head and Neck Surgery, Baylor College of Medicine, Houston, TX, United States

**Keywords:** papillomavirus vaccines, papillomavirus infections, social media, sentiment analysis, COVID-19

## Abstract

**Background:**

Human papillomavirus (HPV) is a sexually transmitted virus that causes various oropharyngeal and anogenital cancers. The HPV vaccine provides protection against several strains of HPV and is a key preventative tool against HPV-related cancers; however, vaccination rates remain suboptimal in the United States due to variable state mandates and misperceptions of vaccine efficacy and risks. As social media becomes an increasingly popular avenue for health discussions, platforms such as Reddit offer opportunities to understand public vaccine discourse, particularly among underrepresented groups. Furthermore, vaccine hesitancy and mistrust generally increased during the COVID-19 pandemic, potentially impacting HPV vaccination rates.

**Objective:**

This study aimed to characterize HPV vaccine–related discussions on Reddit by (1) identifying dominant themes, (2) assessing sentiment, and (3) examining shifts in themes and sentiment before and after the start of the COVID-19 pandemic.

**Methods:**

This convergent mixed methods analysis used a Python-based script to extract Reddit posts and comments referencing the HPV vaccine from multiple subreddits from September 13, 2016, to February 13, 2025. Entries were manually labeled as intentional or incidental, with intentional entries further categorized into 10 thematic categories. Sentiment analysis was conducted using the VADER (Valence Aware Dictionary for Sentiment Reasoning) algorithm. Pre– vs post–COVID-19 comparisons used March 11, 2020, as the cutoff date. Temporal trends were assessed using pre– and post–COVID-19 stratification. Chi-square tests, Mann-Whitney *U* tests, and linear regression were used for statistical analysis.

**Results:**

Of 4235 collected entries, 2801 (66.1%) intentional posts and comments were analyzed. The most common themes were factual claims (703/2801, 25.1%), personal experiences (571/2801, 20.4%), and offering advice (465/2801, 16.6%). Overall sentiment was 51.7% (1448/2801) positive (95% CI 49.8%-53.5%), 37.6% (1053/2801) negative, and 10.8% (300/2801) neutral, with a median sentiment score of 0.13 (IQR –0.49 to 0.71). Pre–COVID-19 entries (n=278) were 124 (44.6%) positive (95% CI 38.7%-50.7%), with a median sentiment of 0.00 (–0.66 to 0.58). Post–COVID-19 entries (n=2523) were 1324 (52.4%) positive (95% CI 50.5%-54.4%), with a median sentiment of 0.17 (–0.46 to 0.72). Sentiment increased significantly post–COVID-19 (*P*=.01). Theme distribution differed before vs after COVID-19 (*χ*²_9_=114.47, *P*<.001). Pre–COVID-19 discussions overrepresented barriers and social or cultural influences, whereas post–COVID-19 discussions more frequently reflected personal experiences, advice seeking, and advice offering. Posting volume increased by 50% per year throughout the study period (incidence rate ratio=1.50, 95% CI 1.47-1.53; *P*<.001), with a steeper increase post–COVID-19 (incidence rate ratio=1.60, 95% CI 1.56-1.65).

**Conclusions:**

This study provides a post–COVID-19 perspective on patient-generated HPV vaccine discourse by integrating thematic content with sentiment and temporal trends. Prior studies have been limited by a narrower thematic scope and have focused on prepandemic periods. By examining discussions on an anonymous platform such as Reddit, this analysis captures personal conversations and assesses pandemic-related shifts in discourse. These findings add to the literature on digital vaccine communication and highlight opportunities for targeted public health engagement and strategies to leverage online communities.

## Introduction

Human papillomavirus (HPV) is a sexually transmitted virus and a well-established risk factor for various cancers in both men and women [1]. Most commonly associated with cervical cancer, HPV has also been increasingly linked to anogenital and oropharyngeal cancers [1,2]. High-risk HPV strains, particularly types 16 and 18, are responsible for over 70% of these cases, while low-risk strains cause benign conditions such as papillomas and warts [3,4].

Vaccines have been developed to protect against high- and low-risk HPV strains, reducing the likelihood of persistent infection. The first HPV vaccine, Gardasil, was approved by the US Food and Drug Administration in 2006 and provided protection against HPV 6, 11, 16, and 18 [5]. Subsequent vaccines, such as Gardasil 9, expanded coverage to include 5 additional high-risk strains (HPV 31, 33, 45, 52, and 58), offering broader protection against HPV-associated cancers [6]. Research has shown that HPV vaccination is highly effective in preventing certain cancers [7]. Despite this, vaccination coverage remains suboptimal, with only about 66% of eligible individuals completing the full vaccine series by age 17 and approximately 78% receiving at least 1 dose [8,9].

Unlike vaccines routinely required for school attendance, the HPV vaccine encounters distinct challenges. Mandates for HPV vaccination exist in only a few states, and even these often include opt-out provisions that hinder widespread uptake [10]. Globally, there is a varying level of support for the HPV vaccine among parents, which is particularly low in the United States. Parents of sons are less likely to promote HPV vaccination for their children or may be unaware of its existence or efficacy [11]. Studies have shown that education on HPV in schools led to a positive impact on parents supporting HPV vaccination for their children [12,13]. Since its introduction, the vaccine has also been embroiled in controversy, with public hesitancy fueled by concerns regarding its safety, efficacy, and perceived association with sexual activity [10,14]. A lack of awareness of HPV infection in men and its effects on them and their sexual partners may contribute to this skepticism, although research has shown that men benefit from HPV vaccination in preventing anogenital disease [15]. Moreover, the COVID-19 pandemic limited the availability of vaccines and coverage [13] and may have exacerbated this skepticism, as heightened public discourse around vaccines amplified misinformation, emotional responses, and confusion [16,17]. Data show that HPV vaccination rates were increasing until 2022 [18], which may have been affected by increased vaccine hesitancy during the COVID-19 pandemic.

While health care providers typically rely on evidence-based guidelines for vaccine advocacy, this approach can sometimes overlook the personal experiences, specific concerns, and misconceptions held by patients. Addressing these individual perspectives is crucial for improving HPV vaccination rates and for bridging the divide between scientific recommendations and public apprehension. Although research exists examining providers regarding HPV vaccine hesitancy based on their experiences with patients, patient perspectives are not well represented in the literature [17]. There are also many groups that may be underrepresented in this understanding of HPV vaccine hesitancy; for instance, there is a paucity of published literature on HPV vaccination among men who have sex with men [2]. In this context, social media platforms provide rich, readily available datasets for exploring patient viewpoints, offering advantages over traditional survey methods, which can be resource-intensive and unable to capture spontaneous, real-time discussions [19].

However, systematically reviewing individual social media posts and comments may hinder the efficacy of research efforts. This is where sentiment analysis is useful, as it is a methodology that uses natural language processing (NLP) to understand the opinions expressed within text and to systemically measure emotional tone [20]. One tool used for sentiment analysis is VADER (Valence Aware Dictionary for Sentiment Reasoning), which has been shown to be useful in deciphering sentiments within social media, such as comments on YouTube [21,22]. VADER operates by using a lexicon or dictionary with predetermined positive, negative, or neutral sentiment values, allowing code to efficiently parse through large amounts of text posted on social media [20].

Although previous research has extensively analyzed HPV vaccine discussions on Twitter, a platform popular among adolescents and young adults who are key demographics for the vaccine, less attention has been paid to corresponding discussions on Reddit [14,19,23-29]. Earlier work by Lama et al [30] examined Reddit discourse from 2007 to 2015 using topic modeling and identified themes such as political debate, sexual behavior, and vaccination schedules. More recent work by Du et al [31] and Meci et al [32] explored misinformation prevalence and conducted sentiment analysis of Reddit posts, respectively, but both studies were limited by narrow thematic scopes and did not assess how discourse evolved in response to the COVID-19 pandemic [31,32]. Recent research by Singh et al [33] evaluated perceptions of HPV vaccination among Indian users on Reddit but was limited to this specific demographic, suggesting that a broader study is necessary.

Consequently, there is a gap in understanding the longitudinal evolution of HPV vaccine discourse on Reddit, particularly regarding shifts in themes and sentiment surrounding major public health events such as the COVID-19 pandemic, analyzed through detailed manual coding. Recent work has shown that VADER is useful in parsing Reddit posts when examining views related to the COVID-19 pandemic [21,34]. This study addresses this gap by analyzing both Reddit posts and comments from 2016 to 2025 using a granular, 10-category manual coding scheme. It uniquely examines how discussion themes and overall sentiment changed before and after the onset of the COVID-19 pandemic.

Reddit offers a distinct environment for such analysis. Unlike platforms often linked to identifiable user accounts, Reddit facilitates anonymous, long-form, asynchronous conversations within topic-specific communities called subreddits. These features foster more candid discussions and deeper exchanges between users. Prior research indicates that Reddit users are predominantly younger than 35 years, with 1 study reporting a mean user age of 25.7 years, which aligns closely with the target demographic for HPV vaccination and makes Reddit particularly relevant for examining vaccine attitudes [31,35]. The large amount of subreddit communities also allows researchers to capture discussions across diverse demographic and behavioral contexts. Because Reddit preserves entire discussion threads and allows posts to resurface through ongoing commenting, it provides a unique opportunity to observe how vaccine-related conversations evolve longitudinally. Analyzing these interactions can illuminate prevalent misconceptions, identify barriers to vaccination, and reveal opportunities for more effective public health communication.

This study aimed to address the following research questions: (1) What key thematic categories characterize Reddit discussions about the HPV vaccine? (2) What is the overall sentiment expressed toward the HPV vaccine on Reddit? and (3) How did themes, sentiment, and discourse change before versus after the start of the COVID-19 pandemic? To answer these questions, we conducted a retrospective cross-sectional analysis of Reddit posts, applying manual thematic categorization and VADER sentiment analysis and comparing temporal patterns prepandemic and post pandemic. Findings from this work may help inform more effective and empathetic communication strategies for health care professionals and public health officials.

## Methods

### Ethical Considerations

This study was reviewed by the Rush University Medical Center Institutional Review Board and was determined to be exempt from human subjects research (5511) due to the secondary use of publicly available data. No informed consent was required because all data were publicly accessible, contained no identifiable private information, and were analyzed in aggregate. No images included in this manuscript or supplementary materials contain identifiable information about individual users. All posts and comments were collected in accordance with Reddit’s application programming interface Terms of Service. All study materials were stored in a secure institutional OneDrive folder. No compensation was provided, as no participants were recruited or directly involved in the study.

### Research Design Overview

This study used a convergent mixed methods descriptive design and followed the APA Journal Article Reporting Standards for mixed methods research [36]. Mixed methods were used to allow the identification of dominant themes in HPV vaccine–related discourse, followed by quantitative analysis of sentiment, thematic frequencies, and temporal trends.

Qualitative and quantitative analyses were conducted on the same set of Reddit posts and comments from September 13, 2016, to February 13, 2025. Qualitative thematic analysis was performed first to classify posts as incidental or intentional HPV vaccine discussions and to assign thematic categories. Quantitative analyses were subsequently conducted to examine thematic distribution, sentiment scores, and posting volume. Integration occurred during analysis and interpretation, with qualitative themes serving as the framework for quantitative comparisons.

### Qualitative Methods

#### Overview

A descriptive qualitative thematic analysis was conducted to identify and characterize patterns of HPV vaccine–related discourse on Reddit.

#### Data Sources and Collection

Posts and comments referencing the HPV vaccine within the study period were collected from multiple subreddits, along with their respective metadata. The subreddits analyzed are outlined in Table 1. Using Visual Studio Code (version 1.90.0 [Universal]), an integrated development environment developed by Microsoft (Multimedia Appendix 1), a data scraping script was written in Python (version 3.9.16) using the PRAW library to interact with the Reddit application programming interface and collect entries containing keywords from selected subreddits to identify those mentioning the HPV vaccine (Table 1). For each post and comment, metadata—including the subreddit name, text body, flair, date of submission, number of upvotes, post or comment ID, parent comment ID, entry type (post or comment), and anonymized author ID—were collected (MultimediaAppendices 2  3).

**Table 1.** Subreddits included in the study and keyword mapping strategy for identifying human papillomavirus vaccine–related posts.^a^

Subreddit category	Subreddits	Keywords applied
HPV^b^-focused	r/HPV, r/PreCervicalCancer, r/Warts, r/CervicalCancer	Vaccine-related keywords: vaccine, vaccines, vaccinated, vaccination, vaccinations, vax, vaxxed, unvaxxed Gardasil, Cervarix anti-vax, antivax, antivaccine, anti-vaccine, pro-vax jab, shot, immunization
Vaccine-focused	r/VACCINES, r/vaxxhappened, r/Vaccine,r/antivax, r/DebateVaccines,r/unvaccinated, r/skeptic, r/StopMandatoryVaccine	HPV-related keywords: HPV, HPV vaccine, HPV shot Gardasil, Cervarix human papillomavirus
Medical and science	r/AskDocs, r/medicine, r/PeterAttia, r/HeadandNeckCancer,r/Health, r/ScienceBasedParenting, r/HNSCC	HPV+vaccine-related keywords: HPV, HPV vaccine, HPV shot, Gardasil, Cervarix, human papillomavirus
Women’s health	r/TwoXChromosomes, r/TheGirlSurvivalGuide, r/WomenOver40, r/AskWomenOver30	HPV+vaccine-related keywords: vaccine, vaccines, vaccinated, vaccination, jab, shot, immunization
General and social	r/MensRights, r/AskGaybrosOver30, r/AskConservatives, r/askgaybros	HPV+vaccine-related keywords: anti-vax, antivax, pro-vax

a This table lists all subreddits analyzed in this retrospective cross-sectional social media content analysis of human papillomavirus vaccine discussions on Reddit. It details each subreddit’s overall category and the keyword strategy applied to identify human papillomavirus vaccine–related posts and comments collected from September 13, 2016, to February 13, 2025.

b HPV: human papillomavirus.

#### Data Selection and Preprocessing

**Figure 1.**
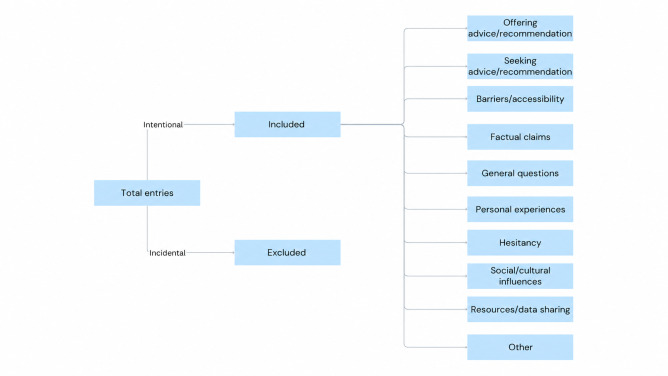
Flowchart of data collection, filtering, and manual labeling of Reddit posts related to the human papillomavirus (HPV) vaccine (2016‐2025). This flowchart summarizes the workflow for identifying and processing Reddit posts and comments referencing the HPV vaccine in this retrospective cross-sectional social media content analysis. After keyword-based extraction (2016‐2025), entries were screened for relevance and classified as intentional (directly discussing HPV vaccination) or incidental (mentioning keywords without substantive relevance and excluded). Intentional entries were then manually coded into 10 mutually exclusive thematic categories for analysis.

Data were collected and preprocessed prior to analysis using the following steps:

1. Keyword mapping: Keywords were divided into 2 primary categories to identify relevant discussion—general vaccine–related terms and HPV-specific terms (Table 1). Keyword groups were mapped to subreddits based on thematic relevance. HPV-specific subreddits (eg, r/HPV) were filtered using the general vaccine–related keyword group, vaccine-focused subreddits (eg, r/VACCINES, r/vaxxhappened) were filtered using the HPV-specific keyword group, and broad medical subreddits (eg, r/AskDocs) used both keyword groups. Keywords were processed to prioritize longer, more specific terms (eg, “HPV vaccine” before “HPV”) to prevent mismatches in which shorter keywords might appear within unrelated longer words or phrases. All retrieved entries and metadata were exported to Microsoft Excel for further processing.
2. Text cleaning and tokenization: Basic NLP preprocessing was performed to prepare entries for analysis. This process consisted of decoding HTML entities, converting all text to lowercase, and tokenization to ensure compatibility with the VADER sentiment tool.
3. Filtering and inclusion: Entries were screened and categorized as either “incidental” or “intentional” discussion. Intentional entries were defined as posts or comments in which the HPV vaccine was the primary focus of discussion. In contrast, incidental entries were defined as content in which a keyword was present but the HPV vaccine was mentioned only tangentially. Examples of incidental entries included listing “HPV vaccine” as part of a general medical history without further discussion or brief references within unrelated discussions in which the vaccine was not the central topic (Figure 1). Inclusion criteria were posts and comments labeled as “intentional” and those consisting of 10 words or more.

#### Thematic Analysis

Intentional entries underwent further classification into 10 thematic subcategories, which are listed and described in Table 2. These 10 categories were created after an initial screening of 200 entries. To ensure reliability, 2 authors (MM, DB) independently performed manual data labeling, and any discrepancies were resolved through discussion and consensus. Because the dataset was predefined and finite, categorization continued until all eligible content was labeled rather than terminating at the point of saturation. Thematic saturation was assessed retrospectively and was considered achieved when no new thematic categories emerged during the later stages of analysis.

**Table 2.** Definitions and examples of thematic categories used in manual coding of Reddit posts.^a^

Theme	Definition	Examples
Advice or recommendations (offering)	Posts that explicitly offer advice or recommendations regarding HPV^b^ vaccination to others.	“You should get vaccinated,” “My doctor recommended getting the vaccine”
Advice or recommendations (seeking)	Posts that request advice or opinions from others about receiving the HPV vaccine.	“Should I get the vaccine?,” “Should I encourage my partner to get the vaccine?”
Barriers or accessibility	Posts that describe challenges in accessing the HPV vaccine, including financial, insurance, geographic, or age-related limitations.	“I wanted the vaccine 3 months before I was 26, insurance said no, my age disqualified coverage, but I hadn’t turned 26 yet,” “My daughter got the shot after she moved out and the first shot was over 400 dollars. I told her about in network and the 2nd was free.”
Factual claims	Statements presented as facts, regardless of accuracy, typically lacking overt opinion or emotion. Intended to convey information or assert knowledge.	“Gardasil 9 protects against nine types of human papillomavirus (HPV): 6, 11, 16, 18, 31, 33, 45, 52, and 58,” “There’s zero data on actual cervical cancer rates declining due to HPV vaccines.”
General questions	Posts that inquire about specific aspects of the HPV vaccine, including dosing, efficacy, safety, or eligibility.	“Is two shots enough to protect you?,” “Why the difference between US CDC 3-dose and UK NHS 2-dose recommendation for HPV vaccination for older adults up to age 45?”
Hesitancy	Posts expressing concerns, skepticism, or fear about HPV vaccine safety, efficacy, or perceived risks. May include conspiracy or mistrust themes.	“I know someone who periodically needs to have her skull drained of excess fluid because of the HPV vaccine. They almost died after taking it due to fluid build up around her brains. I stupidly took it as an adult at 36 and have had autoimmune issues ever since.,” “And my generation, 90% of us have HPV and don’t know it and we’re fine... U think doctors don’t lie? Doctors aren’t corrupt or capable of being corrupted? Just use common sense. Less vacinnes the better IMHO!”
Personal experiences	Narratives sharing personal or family experiences with HPV vaccination, including positive, negative, or neutral outcomes.	“I got my 1st dose of Gardasil vaccine on Thursday. I have a fever of 103.3 and I feel really sick. How are you doing?,” “I was diagnosed with HPV after almost 20 years of marriage... I’m now getting vaccinated at 43, even though I had a hysterectomy because I want to make sure I’m protected from any possible further in the future.,” “I had the vaccine aged 12, and got diagnosed with HPV at my first smear, ended up having a colposcopy to remove dodgy cells. It is not a guaranteed prevention.”
Resources or data sharing	Posts that share articles, external links, or statistical information to support a claim, argument, or question.	“The CDC recommends that women get the HPV vaccine through age 26, if they haven’t already been vaccinated. https://www.cancer.gov/types/cervical/causes-risk-prevention,” “The risk of cancer for low risk strains is quite low. The high risk strains themselves comprise the majority of HPV-derived cancer. See [this table] (https://www.cdc.gov/cancer/hpv/statistics/cases.htm) for a breakdown of risk of cancer type by HPV strain.”
Social or cultural influences	Posts discussing how culture, religion, family, or societal norms influence attitudes or decisions regarding HPV vaccination.	“I thought, because of what the Ministry of Health informed back then in my country, the HPV vaccine only worked in young girls.” “I never got the vaccine when I was younger (due to being too young to make my own decision and my mom being against it). I’m obviously very upset but just happy to see this post and hopefully my HPV clears up and goes dormant once this is all over with”
Other	Posts that do not fit into other categories due to ambiguity, sarcasm, or lack of context.	“At what age did you get the vaccine?,” “I’m not her, so no - her witness testimony is evidence of her claims & she’s more credible than Gardasil.”

a This table summarizes the 10 manually coded thematic categories applied to Reddit posts and comments intentionally discussing the human papillomavirus vaccine. Definitions and representative examples illustrate how each entry was categorized in this cross-sectional analysis of human papillomavirus vaccine discourse on Reddit (2016‐2025).

b HPV: human papillomavirus.

### Quantitative Methods

#### Inclusion and Exclusion Criteria

Analyses of sentiment scores, thematic frequencies, thematic comparisons, and temporal trends were restricted to posts classified as intentional HPV vaccine discussions with 10 words or more. For the analysis of time from a post to the first response, all posts and comments, including both intentional and incidental mentions, were included.

#### Data Sources and Characteristics

Quantitative data consisted of metadata and derived variables from Reddit posts and comments, including posting date, subreddit, upvote counts, response time to first comment, sentiment scores, and assigned thematic categories. Demographic characteristics were unavailable due to the anonymous nature of the platform.

#### Sample Size, Power, and Precision

The final analytic dataset included all posts and comments meeting the inclusion criteria. Given the descriptive and exploratory nature of the study, formal power calculations were not conducted. Precision of estimates was assessed using confidence intervals where applicable.

#### Measures and Data Collection

Sentiment analysis was conducted using the VADER (Valence Aware Dictionary and Sentiment Reasoning) algorithm, which was selected because it is a user-friendly, reliable, and human-validated lexicon-based tool optimized for interpreting sentiment in social media text [22]. VADER assigns each entry a compound score from −1 (most negative) to +1 (most positive). Based on established thresholds, entries were classified as positive (score >0.05), neutral (score between –0.05 and 0.05), or negative (score <–0.05).

Additional quantitative variables were derived from metadata collected at the time of data extraction and from theme frequency counts generated through the qualitative thematic categorization described above.

#### Analytic Strategy

Sentiment scores were analyzed across the entire dataset and within each thematic category. Due to the nonnormal distribution of these scores, the overall sentiment trend was summarized using the median and IQR. However, mean sentiment scores were reported for individual themes to facilitate interpretation and cross-theme comparisons.

To assess the potential impact of the COVID-19 pandemic, entries were temporally stratified based on the WHO pandemic declaration date (March 11, 2020). Entries posted before this date were designated pre–COVID-19, whereas those posted on or after this date were designated post COVID-19. Posting volume per year was also determined. User engagement was assessed by calculating the median response time (time elapsed from submission of an original post to the first collected comment) across all posts within the included subreddits, including both intentional and incidental entries.

Chi-square tests of independence were used to compare theme frequencies between the pre– and post–COVID-19 periods. Standardized residuals (SRs >|2|) were used to identify themes that were significantly overrepresented or underrepresented in each period. Temporal trends in annual posting volume were modeled using Poisson regression, with additional models stratified by pre– vs post–COVID-19 periods. Given the nonparametric distribution of sentiment scores and variability in group sizes, Mann-Whitney *U* tests were used to compare median sentiment overall and within each theme before vs after the pandemic. A linear regression model assessed whether thematic category predicted sentiment score, using “Factual Claims” as the reference due to its prevalence and informational neutrality.

There were no missing data in the final analytic dataset; therefore, no missing completely at random testing or imputation was required. All analyses were conducted in RStudio (version 2024.12.1+563), and statistical significance was defined as *P*<.05.

## Results

### Overview

A total of 4235 Reddit entries referencing the HPV vaccine were collected between September 13, 2016, and February 13, 2025. Of those, 2803 were classified as intentional posts or comments. After excluding 2 entries from 2011 that fell outside the defined study period, 2801 intentional entries were included in the final analysis, including 614 posts and 2187 comments.

Factual claims were most common (703/2801, 25.1%), followed by personal experiences (573/2801, 20.4%) and advice offering (466/2801, 16.6%) (Table 3). Overall, 1447 of 2801 (51.7%) entries exhibited positive sentiment (95% CI 49.8%‐53.5%), 1052 of 2801 (37.6%) negative, and 302 of 2801 (10.8%) neutral. The median sentiment score across all entries was 0.13 (IQR –0.49 to 0.71). At the theme level, advice seeking had the highest mean sentiment (0.17, SD 0.62), whereas “Other” entries were slightly negative (–0.04, SD 0.65).

**Table 3.** Frequencies of thematic categories and mean sentiment scores in Reddit discussions about the human papillomavirus vaccine before and after the World Health Organization COVID-19 pandemic declaration (2016‐2025).^a^

Theme	Pre–COVID-19, n (%)^b^	Pre–COVID-19 sentiment, mean (SD)	Post–COVID-19, n (%)	Post–COVID-19 sentiment, mean (SD)	Total, n (%)	Total sentiment, mean (SD)
Advice or recommendations (offering)	34 (12.2)	0.05 (0.69)	432 (17.1)	0.12 (0.63)	466 (16.6)	0.12 (0.64)
Advice or recommendations (seeking)	5 (1.8)	−0.09 (0.30)	148 (5.9)	0.18 (0.63)	153 (5.5)	0.17 (0.62)
Barriers or accessibility	52 (18.7)	0.03 (0.71)	168 (6.7)	0.12 (0.63)	220 (7.8)	0.10 (0.65)
Factual claims	71 (25.5)	0.00 (0.62)	632 (25)	0.09 (0.65)	703 (25.1)	0.08 (0.65)
General questions	16 (5.8)	−0.01 (0.56)	199 (7.9)	0.08 (0.69)	215 (7.7)	0.07 (0.68)
Hesitancy	9 (3.2)	−0.27 (0.52)	132 (5.2)	0.09 (0.65)	141 (5)	0.07 (0.64)
Personal experiences	37 (13.3)	0.08 (0.62)	536 (21.2)	0.14 (0.64)	573 (20.4)	0.13 (0.64)
Resources or data sharing	21 (7.6)	0.05 (0.70)	161 (6.4)	0.10 (0.66)	182 (6.5)	0.09 (0.67)
Social or cultural influences	28 (10.1)	−0.17 (0.73)	62 (2.5)	0.14 (0.68)	90 (3.2)	0.04 (0.71)
Other	5 (1.8)	0.11 (0.67)	53 (2.1)	−0.05 (0.65)	58 (2.1)	−0.04 (0.65)
Total	278	0.00 (0.65)	2523	0.11 (0.65)	2801	0.10 (0.65)

a This table reports the total number of entries within each of the ten manually coded themes, along with theme-specific frequencies and mean (SD) sentiment scores stratified by time period, in a retrospective cross-sectional social media content analysis of Reddit posts and comments. Data include intentional human papillomavirus vaccine–related entries collected from publicly available Reddit discussions between September 2016 and February 2025 (N=2801). Pre–COVID-19 entries were defined as those posted before March 11, 2020, and post–COVID-19 entries as those posted on or after this date.

b Counts and percentages represent the total number of entries per theme in each period. Mean sentiment scores (SD) are shown alongside their respective theme frequencies.

### Pre– vs Post–COVID-19 Volume, Themes, and Sentiment

**Figure 2.**
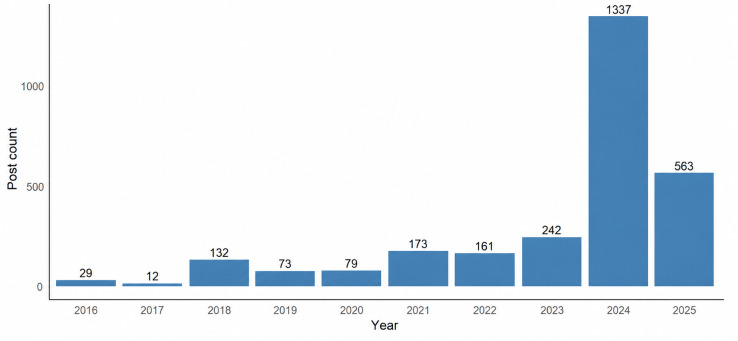
Annual volume of Reddit posts and comments intentionally referencing the human papillomavirus (HPV) vaccine, September 2016-February 2025. This figure depicts the yearly count of publicly available Reddit posts and comments containing HPV vaccine–related keywords collected for this retrospective cross-sectional social media content analysis. Data include intentional entries collected from multiple subreddits between September 13, 2016, and February 13, 2025. Bars represent the total annual volume of intentional entries. A sharp increase in activity is observed beginning in 2021 and peaking in 2024, reflecting growing online engagement with HPV vaccine discourse over time. Importantly, counts for 2025 include only data through February 13 and already approach 600 entries, indicating that activity is on pace to surpass 2024 by the end of the year.

Temporal analysis revealed increasing engagement with HPV vaccine topics over time (Figure 2). Annual post counts rose from 12 in 2016 to 132 in 2018 and then accelerated after 2021, peaking at 1337 in 2024 (Figure 2). Early 2025 data included 563 entries through February 13, suggesting that posting volume in 2025 is on pace to surpass 2024. A Poisson regression model assessing annual post and comment volume from 2016 to 2025 confirmed a significant upward trend. Each additional year was associated with a log-linear increase in count (*β*=.41, SE=0.01; IRR=1.50, 95% CI 1.47‐1.53; *P*<.001), corresponding to a 50% increase in activity per year. When stratified, the pre–COVID-19 period showed a 13% annual increase (*β*=.12, SE=0.04; IRR=1.13, 95% CI 1.04‐1.23; *P*=.01), while the post–COVID-19 period demonstrated a steeper 60% annual increase (*β*=.47, SE=0.01; IRR=1.60, 95% CI 1.56‐1.65; *P*<.001).

Of the 2801 intentional entries, 278 occurred pre–COVID-19 (September 2016 to March 2020) and 2523 post–COVID-19 (April 2020 to February 2025). Theme distributions differed significantly pre– vs post–COVID-19 periods (*χ*²_9_=114.47, *P*<.001). SRs indicated that prepandemic entries were overrepresented in barriers or accessibility (SR=7.09) and social or cultural influences (SR=6.83), whereas postpandemic entries were overrepresented in personal experiences (SR=3.11), advice seeking (SR=2.83), and advice offering (SR=2.08).

Sentiment also differed across periods. Pre–COVID-19 entries were 44.6% (124/278) positive (95% CI 38.7%‐50.7%), 43.9% (122/278) negative, and 11.5% (32/278) neutral sentiment, with a median score of 0.00 (–0.66 to 0.58). Post–COVID-19 sentiment saw an increase in positive sentiment, with entries being 52.4% (1324/2523) positive (95% CI 50.5%‐54.4%), 36.9% (931/2523) negative, and 10.7% (268/2523) neutral, with a median score of 0.17 (IQR –0.46 to 0.72). A Mann-Whitney *U* test confirmed a statistically significant increase in sentiment scores post–COVID-19 (W=384615, *P*=.01), suggesting a shift toward a more positive tone in HPV vaccine discussions since the onset of the pandemic.

When sentiment changes were assessed within individual themes, no category-specific comparisons reached statistical significance (all *P*>.05). However, some themes demonstrated notable shifts. For example, vaccine hesitancy changed from a mean of –0.27 to +0.09 (*P*=.09), and social or cultural influences shifted from –0.17 to 0.14 (*P*=.08). Although these changes were not statistically significant, they may indicate evolving tone and engagement, possibly reflecting more constructive or reflective dialog in the post–COVID-19 period.

### Linear Regression of Themes Predicting Sentiment Scores

The linear regression model examining theme as a predictor of sentiment score was not statistically significant (*F*_9,2791_=0.98, *P*=.45), indicating that thematic category did not significantly predict sentiment score (*R*²=0.003) when controlling for other themes using “Factual claims” as the reference category. No individual theme coefficient was statistically significant.

### Median Response Time

To assess user interaction and determine whether Reddit serves as a reliable platform for receiving timely responses to vaccine-related inquiries, we analyzed the median time from post to first comment. The overall median response time was 136.1 (IQR 36.0-613.1) minutes, suggesting that users typically received replies within a few hours. However, engagement varied by subreddit. Among the most active communities, r/HPV (850 unique posts) had a median response time of 148.2 (IQR 36.0-613.6) minutes, r/PreCervicalCancer (110 posts) had a response time of 215.6 (IQR 44.4-1184.1) minutes, and r/VACCINES (102 posts) showed a faster response time of 109.8 (IQR 36.1-501.9) minutes. Additional subreddits included r/Vaccine (29 posts), with a median time of 310.1 (IQR 62.1-1053.4) minutes; r/CervicalCancer (18 posts), with a median response time of 94.1 (IQR 37.3-180.6) minutes; r/DebateVaccines (17 posts), with a median response time of 115.7 (IQR 21.3-472.0) minutes; r/vaxxhappened (13 posts), with a median response time of 417.9 (IQR 74.3-748.3) minutes; and r/AskDocs (10 posts), with a median response time of 77.6 (IQR 54.6-80.0) minutes. Subreddits with fewer than 10 unique posts are summarized in Table 1 and were excluded from direct comparisons due to small sample size. This response time analysis included all collected entries, including both intentional and incidental entries, for posts initiating threads.

## Discussion

### Limitations

This study analyzed 2801 Reddit posts and comments to characterize public discourse surrounding the HPV vaccine, identify dominant themes and sentiment, and evaluate how these patterns changed following the start of the COVID-19 pandemic. Factual claims, personal experiences, and advice offering were the most common themes, and overall sentiment was more positive than negative, with a median sentiment score of 0.13. Posting volume increased substantially over time, rising by 50% per year overall and more rapidly after 2020. Post–COVID-19 entries demonstrated significantly higher positive sentiment and a shift toward more advice seeking and personal experience–based discussions. Confidence interval patterns aligned with sample size distribution, with estimates for the pre–COVID-19 period being less precise due to smaller sample counts, whereas overall and post–COVID-19 estimates had narrow confidence intervals, reflecting high precision and more stable inference. These findings indicate that HPV vaccine conversations on Reddit have become more frequent, more positive in tone, and increasingly peer support driven.

Additionally, with an overall median post-to-first-comment time of just over 2 hours and subreddit communities with member counts in the tens or hundreds of thousands, users can quickly ask questions and receive feedback. This responsiveness is particularly relevant in the post–COVID-19 era, during which patient portal messages increased by 157%, potentially causing delays in direct communication with health care providers [37]. The marked rise in HPV vaccine discussions after 2020 (Figure 2) may therefore reflect a growing demand for timely, community-driven health information amid pandemic-related uncertainty. Because Reddit engagement is high and sentiment is increasingly supportive, it represents a critical and underutilized method for targeted interventions designed to address vaccine hesitancy, spread accurate information, and improve HPV vaccination uptake.

The COVID-19 pandemic created heightened vaccine awareness and public debate [38], which likely shaped the tone and content of HPV vaccine discussions observed in this study. While some studies report a decline in vaccine confidence post–COVID-19 [39], a recent literature review found increased uptake of routine adult vaccinations, such as influenza and pneumococcal vaccines, alongside decreased childhood vaccination rates [40]. Similarly, a cross-sectional survey showed that most participants had no change in opinion regarding the HPV vaccine following the pandemic [41]. Together, these contrasting findings underscore the complex and evolving nature of public attitudes toward vaccination across populations and vaccine types. The broad discussions surrounding vaccines likely influenced the volume and sentiment of discussions in this study. Heightened public uncertainty surrounding vaccines may have prompted more users to seek answers and advice on Reddit, along with other social media [42]. The increase in sentiment observed in the study was somewhat unexpected, as heightened skepticism and polarization during the pandemic might have suggested a decline in favorable attitudes [42]. One explanation is that oppositional views may have been redirected toward COVID-specific subreddits during this time, leaving HPV-focused discussions to adopt a more supportive and informational tone. Additionally, users in these subreddits may have felt more supportive and engaged in more constructive discussions surrounding the vaccine during a period marked by broader health uncertainty.

The shift toward more experience-driven content and increasingly positive sentiment may point to Reddit’s growing role as an informal support network and a disseminator of health information [43]. The results suggest that individuals use the platform not only to access information but also to validate their concerns, share lived experiences, and support others navigating vaccine decisions. This trend is further reflected in the growing volume of HPV vaccine–related posts over time. Poisson regression modeling showed a 50% annual increase in post volume from 2016 to 2025, with a sharper rise post–COVID-19 (60% per year) compared to pre–COVID-19 (13% per year). However, these findings should be interpreted alongside broader changes in the platform itself. Reddit’s user demographics, community structures, and overall engagement have evolved considerably over this period [35]. External sociopolitical events, such as the 2020 US presidential election, which coincided near our pandemic cutoff, may also have influenced online vaccine discourse [42]. Considering these factors, public health professionals may benefit from monitoring Reddit and similar platforms to identify emerging concerns, counter misinformation, and tailor outreach strategies to align with community-driven dialog.

However, the unmoderated nature of many social media discussions allows misinformation to spread rapidly. For instance, a prior 2021 study identified misinformation in 25.6% of HPV vaccine–related Reddit posts [31]. These falsehoods can complicate public health messaging and create confusion among users. More studies similar to the work of Du et al [31] should explore subreddit-specific misinformation trends to better understand the nature of these inaccuracies and to support the development of targeted interventions. For example, Reddit users often frame their concerns in personal or culturally specific terms [44]. Public health campaigns may benefit from tailored messaging that acknowledges these concerns and shares relatable narratives or culturally competent information [44]. Interventions might focus especially on younger adults, who make up a large proportion of Reddit users and who may still be within vaccine-eligible age groups [35].

Another notable finding was the shift in sentiment within the vaccine hesitancy and social or cultural influence themes. Although not statistically significant, the directional trend may still hold public health relevance. Both categories shifted from negative sentiment pre–COVID-19 to positive sentiment post–COVID-19, resulting in an overall neutral average. Although individual posts were not examined in detail, this shift may suggest a transition from overt skepticism to more reflective or constructive dialog. For example, users may have become more likely to express uncertainty rather than opposition, share stories about overcoming hesitancy, or describe how they navigate familial or cultural pressures. These evolving dynamics within Reddit HPV discussions suggest a possible shift toward more open and solution-oriented conversations. While this trend warrants further investigation across other platforms, it represents a meaningful opportunity for targeted public health messaging.

Interestingly, the proportion of positive sentiment (n=1447, 51.7%) approximates recent US HPV vaccination rates, with 66% of adolescents completing the full vaccine series by age 17 [8]. Although demographic data were not collected in this study, prior research shows that Reddit users are predominantly younger than 35 years, with one study citing a mean user age of 25.7 years, and most reside in English-speaking countries [31]. This demographic overlap strengthens the case for using Reddit as a window into the attitudes of vaccine-eligible populations and underscores its value in identifying motivators or barriers to vaccine uptake.

Our findings expand on prior research examining HPV vaccine discourse online. Lama et al [30] analyzed Reddit posts from 2007 to 2015 and identified dominant topics such as political debate, sexual behavior, and vaccination schedules using automated topic modeling. While their study provided early insights into Reddit-based vaccine discussions, it did not assess sentiment or examine temporal shifts in discourse. In addition, Du et al [31] focused specifically on identifying misinformation related to the HPV vaccine on Reddit, offering insight into the prevalence of inaccurate claims within public discourse but without thematic categorization or sentiment analysis.

More recently, Meci et al [32] examined sentiment in Reddit posts but limited their analysis to only 3 broad themes—factual, personal, and conspiracy—and excluded user comments. Their study focused on discourse surrounding US Food and Drug Administration guidance encouraging HPV vaccination for head and neck cancer prevention. In contrast, the present study analyzed both posts and comments, employed manual coding across 10 distinct themes for greater granularity, and specifically investigated temporal shifts in theme distribution and sentiment surrounding the COVID-19 pandemic. This multifaceted approach offers a more nuanced and contemporary understanding of online vaccine discourse.

Overall, this study provides an innovative post–COVID-19 examination of public HPV vaccine discourse by integrating qualitative thematic analysis with quantitative sentiment and temporal trends on Reddit. Unlike prior studies that relied on prepandemic data or narrower thematic scope, this analysis offers a more detailed and contemporary view by examining both posts and comments across 10 distinct themes using manual labeling to enhance contextual accuracy. The findings demonstrate a shift toward more personal, advice-oriented, and supportive engagement following the pandemic, contributing to the growing literature on digital vaccine communication. Practically, these findings highlight the value of social media platforms such as Reddit for targeted public health outreach, identification of emerging concerns, and strategies to counter misinformation while supporting efforts to promote HPV vaccine uptake.

### Limitations

This study has several limitations. First, selection and reporting bias are inherent to social media research, which limits generalizability. Although prior research suggests that Reddit users are predominantly younger than 35 years and primarily located in English-speaking countries, the platform’s anonymity makes it difficult to draw definitive conclusions about user demographics [31,40]. Additionally, while this age group overlaps with key HPV vaccine–eligible populations, the findings may not fully capture the sentiments of all decision-makers, such as parents of adolescents or older adults up to age 45, potentially limiting the transferability of conclusions to these specific subpopulations. Second, Reddit is a worldwide platform, and some discussions may reference non-US health care systems. Countries may vary in the amount of discussion on the HPV vaccine, and differences in background and access barriers may shape discussions in ways that do not reflect the US context. These differences reduce external validity and limit generalizability to US populations. Third, the study’s cross-sectional and retrospective design limits causal inference. User perspectives cannot be followed longitudinally, and shifts in discussion cannot be attributed to specific events with certainty. Additionally, some subreddits archive older posts, which may have contributed to the lower volume of retrieved entries during the earlier years of the study period. Fourth, thematic categorization required assigning each entry to a single dominant theme, which may oversimplify entries containing multiple topics. Although 2 independent coders resolved discrepancies by consensus to maximize consistency and reliability, some nuances may inevitably be lost. Finally, the reliance on an automated tool (VADER) for sentiment analysis presents limitations. While efficient for large datasets and social media, automated scoring may fail to capture sarcasm, medical jargon, context-dependent nuances, or complex emotional expressions accurately.

### Future Directions

Future research could employ more advanced NLP techniques (eg, topic modeling within themes and stance detection) to further dissect nuanced discussion threads. This approach could enable more fine-grained identification of specific concerns, misinformation patterns, or information gaps within each theme. In turn, such insights could better inform public health campaigns and digital outreach efforts aimed at improving vaccine uptake, trust, and health literacy in online spaces.

Additionally, future studies could explore whether awareness of HPV-related oropharyngeal cancers, particularly in men, is increasing over time through social media discourse. Prior survey research has documented low public awareness of the link between HPV and oropharyngeal cancer in men [45]. Because HPV is often associated with cervical cancer, many men may not perceive the vaccine as relevant to them, contributing to lower uptake and greater vaccine hesitancy in this group. A broader assessment of these knowledge gaps across social media platforms could support the development of targeted, gender-specific educational campaigns. Tailored messaging that addresses misconceptions and highlights the relevance of HPV vaccination for men may be critical to improving coverage and informed decision-making in vaccine-hesitant populations.

### Conclusion

This study provides a contemporary analysis of HPV vaccine discourse and sentiment on Reddit, highlighting significant shifts in both thematic focus and sentiment before and after the COVID-19 pandemic. Findings suggest that conversations have become increasingly personal and advice-driven over time, with a notable rise in positive sentiment and public engagement following 2020. These trends reflect a growing reliance on online communities for health-related dialogue and peer support, particularly within a digital landscape transformed by the pandemic. Reddit appears to serve not only as a platform for information exchange but also as a space for collective decision-making, emotional validation, and rapid feedback, especially in the context of health care system delays and uncertainty. These insights underscore the importance of public health professionals monitoring and potentially engaging with evolving online conversations to promote accurate vaccine information and respond to emerging concerns in real time. This work builds on prior research while offering new perspectives on the dynamic, community-driven nature of digital health communication in the social media era.

## Supplementary material

10.2196/83558 Multimedia Appendix 1 Microsoft Excel spreadsheet containing all posts and comments scraped, along with their labels and sentiment scores.

10.2196/83558 Multimedia Appendix 2 Natural language processing code used for sentiment analysis.

10.2196/83558 Multimedia Appendix 3 Python script used to scrape data from Reddit.

## Abbreviations

HPV: human papillomavirus
NLP: natural language processing
SR: standardized residual
VADER: Valence Aware Dictionary and Sentiment Reasoner
