# Supplementary material for: Human Papillomavirus Vaccine Discourse and Sentiment on Reddit Before and After COVID-19: Mixed Methods Retrospective Cross-Sectional Study
Source: J Med Internet Res. 2026 May 19;28:e83558. doi: 10.2196/83558 (PMC13186556; doi:10.2196/83558)
Supplement: Multimedia Appendix 2 [file jmir-v28-e83558-s002.pdf]

```
import pandas as pd
import os
import matplotlib.pyplot as plt
from vaderSentiment.vaderSentiment import SentimentIntensityAnalyzer
from sklearn.feature_extraction.text import TfidfVectorizer
from sklearn.decomposition import LatentDirichletAllocation, TruncatedSVD
import text2emotion as te
from openpyxl import load_workbook

# --------------------------------------------------------------------------------
# 🗁 Input and Output Paths
# --------------------------------------------------------------------------------
file_path = '/Users/Sean/Library/CloudStorage/OneDrive-rush.edu/Rush Research
Projects/Reddit Projects/HPV Vaccine Perceptions/HPV Manuscript Data.xlsx'
figures_dir = '/Users/Sean/Library/CloudStorage/OneDrive-rush.edu/Rush Research
Projects/Reddit Projects/HPV Vaccine Perceptions/NLP_Figures'
os.makedirs(figures_dir, exist_ok=True)
print("🗁 File paths and directories set up.")

# --------------------------------------------------------------------------------
# ◈ Load Data from "Scraped Data" Sheet
# --------------------------------------------------------------------------------
def load_existing_data(file_path):
    print("📥 Attempting to load existing data...")
    return pd.read_excel(file_path, sheet_name='Scraped Data') if
os.path.exists(file_path) else pd.DataFrame()

df = load_existing_data(file_path)

if df.empty or "Original Text" not in df.columns:
    print("❌ Error: No existing data or 'Original Text' column missing.")
else:
    print("✅ Data loaded successfully. Processing...")

    # --------------------------------------------------------------------------------
    # 1. SENTIMENT ANALYSIS (VADER)
    # --------------------------------------------------------------------------------
    print("◍ Performing Sentiment Analysis...")

    sia = SentimentIntensityAnalyzer()

    def compute_sentiment(text):
        # Returns the full polarity dictionary, e.g. {'neg': 0.0, 'neu': 0.5, 'pos':
0.5, 'compound': 0.5}
        return sia.polarity_scores(text)

    def get_sentiment_label(compound):
        if compound >= 0.05:
            return "Positive"
        elif compound <= -0.05:
```

```
            return "Negative"
        else:
            return "Neutral"

    # Create new sentiment columns based on "Original Text"
    df["Sentiment Compound Score"] = df["Original Text"].fillna("").apply(lambda x:
compute_sentiment(x)["compound"])
    df["Sentiment Label"] = df["Sentiment Compound
Score"].apply(get_sentiment_label)
    print("✅ Sentiment Analysis completed.")

  import pandas as pd
import re
import html
import time
import json # Import json library again
from vaderSentiment.vaderSentiment import SentimentIntensityAnalyzer
from transformers import pipeline
from transformers.utils import logging as hf_logging
import text2emotion as te
import warnings

# --- Configuration ---
INPUT_FILE = '/Users/Sean/Library/CloudStorage/OneDrive-rush.edu/Rush Research
Projects/Reddit Projects/Head and Neck Cancer/HNC_Abstract_Data.xlsx'
# Updated output name
OUTPUT_FILE = '/Users/Sean/Library/CloudStorage/OneDrive-rush.edu/Rush Research
Projects/Reddit Projects/Head and Neck
Cancer/HNC_Abstract_Data_with_T3Str_Emotion.xlsx'
SHEET_NAME = 'Scraped Data'
TEXT_COL = 'Original Text'
LAST_EXISTING_COL = 'Treatment_Modalities'

# New column names (in order of insertion)
NEW_COL_CLEANED_V2 = 'Pre-Processed v2'
NEW_COL_CLEANED_V2_WC = 'Pre-Processed v2 Word Count'
NEW_COL_VADER_SCORE = 'VADER Score'
NEW_COL_VADER_LABEL = 'VADER Label'
NEW_COL_T2E_SCORES = 't2e Emotion Scores Dict'
NEW_COL_T2E_LABEL = 't2e Dominant Emotion'
NEW_COL_TRANSFORMER_SENT_LABEL = 'HF Sentiment Label'
NEW_COL_TRANSFORMER_SENT_SCORE = 'HF Sentiment Score'
# New columns for HF Emotion model (Top 3 String + Dominant)
NEW_COL_HF_EMOTION_TOP3_STR = 'HF Emotion Top3 (JSON)' # Stores Top 3 list as JSON
string
NEW_COL_HF_EMOTION_DOMINANT = 'HF Dominant Emotion'   # Label with highest score

# Transformer models
TRANSFORMER_MODEL_SENTIMENT = "cardiffnlp/twitter-roberta-base-sentiment-latest"
TRANSFORMER_MODEL_EMOTION =
```

```
"kashyaparun/Mental-Health-Chatbot-using-RoBERTa-fine-tuned-on-GoEmotion"

# Batch size
BATCH_SIZE = 16

# Suppress warnings/logs
hf_logging.set_verbosity_error()
warnings.filterwarnings("ignore", category=UserWarning, module='openpyxl')
warnings.filterwarnings("ignore", category=FutureWarning)

# --- Pre-processing Function (VADER-friendly) ---
URL_PAT = re.compile(r'https?://\S+|www\.\S+')
MENTION_PAT = re.compile(r'u/[A-Za-z0-9_-]+|/u/[A-Za-z0-9_-]+|@[A-Za-z0-9_-]+')
def clean_for_vader(text: str) -> str:
    # ... (same as before) ...
    if not isinstance(text, str): return ""
    try: text = html.unescape(text)
    except Exception: pass
    text = URL_PAT.sub("<URL>", text)
    text = MENTION_PAT.sub("<USER>", text)
    text = text.replace("\n", " ")
    text = re.sub(r"\s+", " ", text).strip()
    return text

# --- VADER Analysis Function ---
vader_analyzer = SentimentIntensityAnalyzer()
def get_vader_sentiment(text: str) -> tuple:
    # ... (same as before) ...
    if not isinstance(text, str) or not text.strip(): return None, "Neutral"
    try:
        vs = vader_analyzer.polarity_scores(text)
        compound_score = vs['compound']
        if compound_score >= 0.05: label = "Positive"
        elif compound_score <= -0.05: label = "Negative"
        else: label = "Neutral"
        return compound_score, label
    except Exception as e: return None, "Error"

# --- text2emotion Functions ---
def compute_emotion_scores(text):
    # ... (same as before) ...
    if not isinstance(text, str) or not text.strip(): return {}
    try: return te.get_emotion(text)
    except Exception as e: return {"Error": 1.0}

def get_dominant_emotion(emotion_dict):
    # ... (same as before) ...
    if not emotion_dict or "Error" in emotion_dict or all(score == 0 for score in
emotion_dict.values()):
        if "Error" in emotion_dict: return "Error"
```

```
        return "Neutral/None"
    try: return max(emotion_dict, key=emotion_dict.get)
    except Exception: return "Error"

# --- Transformer Pipelines Initialization ---
sentiment_pipeline = None
emotion_pipeline = None
# ... (Same loading logic) ...
print(f"Loading transformer model (Sentiment): {TRANSFORMER_MODEL_SENTIMENT}...")
try:
    sentiment_pipeline = pipeline("sentiment-analysis",
model=TRANSFORMER_MODEL_SENTIMENT)
    print("Sentiment model loaded successfully.")
except Exception as e:
    print(f"Error loading sentiment model: {e}. Sentiment analysis will not be
performed.")

print(f"Loading transformer model (Emotion - GoEmotions based):
{TRANSFORMER_MODEL_EMOTION}...")
try:
    emotion_pipeline = pipeline("text-classification",
model=TRANSFORMER_MODEL_EMOTION)
    print("Emotion model loaded successfully.")
except Exception as e:
    print(f"Error loading emotion model: {e}. Emotion analysis will not be
performed.")


# --- Transformer Batch Processing Function ---
# Modified to return Top 3 as JSON string + Dominant Label
def get_transformer_analyses_batch(texts: list) -> tuple:
    processed_texts = [str(text) if pd.notna(text) else "" for text in texts]
    batch_len = len(texts)

    # Initialize results lists
    sent_labels, sent_scores = ["Error"] * batch_len, [None] * batch_len
    # Initialize lists for the new emotion output format
    emot_top3_scores_str = ["N/A"] * batch_len # Store JSON string here
    emot_dominant_labels = ["N/A"] * batch_len # Store dominant label here

    # Sentiment Analysis
    if sentiment_pipeline:
        try:
            with warnings.catch_warnings(): warnings.simplefilter("ignore")
            sentiment_results = sentiment_pipeline(processed_texts, truncation=True,
max_length=512)
            for i, result in enumerate(sentiment_results):
                 if isinstance(result, dict) and 'label' in result and 'score' in
result:
                    sent_labels[i] = result['label']
```

```
                    sent_scores[i] = result['score']
                 else: sent_labels[i] = "Format Error"
        except Exception as e: print(f"Batch sentiment processing error: {e}")

    # Emotion Analysis (Top 3 Scores String + Dominant Label)
    if emotion_pipeline:
        try:
            with warnings.catch_warnings(): warnings.simplefilter("ignore")
                 # Request top 3 results
                 emotion_results_top3 = emotion_pipeline(processed_texts,
truncation=True, max_length=512, top_k=3) # Use top_k=3

            # Output is typically: [[{'label': 'L1', 'score': S1}, {'label': 'L2',
'score': S2}, ...], ...]
            for i, result_list in enumerate(emotion_results_top3):
                 if isinstance(result_list, list) and len(result_list) > 0:
                     # Store the list of top 3 dictionaries as a JSON string
                     try:
                         emot_top3_scores_str[i] = json.dumps(result_list)
                     except Exception as json_e:
                         print(f"Error converting emotion results to JSON:
{json_e}")
                         emot_top3_scores_str[i] = '{"Error": "JSON Conversion
Failed"}'

                     # Extract the dominant emotion (label of the first item)
                     if isinstance(result_list[0], dict) and 'label' in
result_list[0]:
                         emot_dominant_labels[i] = result_list[0]['label']
                     else:
                          emot_dominant_labels[i] = "Format Error"
                 else:
                     # Handle cases where the model returns unexpected format or
empty list
                     emot_top3_scores_str[i] = '[]' # Empty list as JSON
                     emot_dominant_labels[i] = "No Prediction"

        except Exception as e:
            print(f"Batch emotion processing error: {e}")
            emot_top3_scores_str = ['{"Error": "Processing Failed"}'] * batch_len
            emot_dominant_labels = ["Error"] * batch_len


    # Return the 4 relevant lists
    return (sent_labels, sent_scores,
            emot_top3_scores_str, emot_dominant_labels)


# --- Main Script Logic ---
def main():
```

```
    start_time = time.time()
    # --- Initial Notes ---
    # ... (Same notes as before) ...
    print("--- Analyses Performed ---")
    print("1. VADER Sentiment (on Cleaned Text)")
    print("2. text2emotion Emotion (on Original Text)")
    print("3. Transformer Sentiment (on Original Text)")
    print("4. Transformer Emotion (Top 3 Scores JSON + Dominant Label) (on Original
Text)")
    print("--- Note ---")
    print("The model 'mental/mental-bert-base-uncased' is not included.")
    print("----------------------------------")

    # --- Read Data ---
    # ... (Same read logic) ...
    print(f"\nReading Excel file: {INPUT_FILE}, Sheet: {SHEET_NAME}")
    try:
        df = pd.read_excel(INPUT_FILE, sheet_name=SHEET_NAME, engine="openpyxl")
        print(f"Successfully read {len(df)} rows.")
        if TEXT_COL not in df.columns: print(f"Error: Column '{TEXT_COL}' not
found."); return
        if LAST_EXISTING_COL not in df.columns:
             print(f"Warning: Anchor column '{LAST_EXISTING_COL}' not found. New
columns appended at the end.")
             insert_loc = len(df.columns)
        else: insert_loc = df.columns.get_loc(LAST_EXISTING_COL) + 1
    except FileNotFoundError: print(f"Error: Input file not found at {INPUT_FILE}");
return
    except Exception as e: print(f"Error reading Excel file: {e}"); return

    # --- VADER Analysis (Step 1) ---
    # ... (Same VADER logic) ...
    print("\nRunning VADER Analysis...")
    print("  Applying VADER pre-processing...")
    df[NEW_COL_CLEANED_V2] = df[TEXT_COL].apply(clean_for_vader)
    print("  Calculating word counts...")
    df[NEW_COL_CLEANED_V2_WC] = df[NEW_COL_CLEANED_V2].apply(lambda x:
len(x.split()) if isinstance(x, str) and x.strip() else 0)
    print("  Running VADER sentiment...")
    vader_results = df[NEW_COL_CLEANED_V2].apply(get_vader_sentiment)
    df[NEW_COL_VADER_SCORE] = vader_results.apply(lambda x: x[0])
    df[NEW_COL_VADER_LABEL] = vader_results.apply(lambda x: x[1])
    print("VADER analysis complete.")

    # --- text2emotion Analysis (Step 2) ---
    # ... (Same text2emotion logic) ...
    print("\nRunning text2emotion Analysis (using 'Original Text')...")
    emotion_scores_list = []
    num_rows_main = len(df)
    for i, text in enumerate(df[TEXT_COL].fillna(""), start=1):
```

```
        emotion_scores_list.append(compute_emotion_scores(text))
        if i % 50 == 0: print(f"  text2emotion: Processed {i} / {num_rows_main}
entries")
    if len(emotion_scores_list) != num_rows_main:
        print(f"Warning: text2emotion list length mismatch
({len(emotion_scores_list)} vs {num_rows_main})")
        emotion_scores_list.extend([{}] * (num_rows_main -
len(emotion_scores_list)))
    df[NEW_COL_T2E_SCORES] = [str(score_dict) for score_dict in emotion_scores_list]
    df[NEW_COL_T2E_LABEL] = [get_dominant_emotion(score_dict) for score_dict in
emotion_scores_list]
    print("text2emotion analysis complete.")


    # --- Transformer Analyses (Step 3) ---
    if sentiment_pipeline or emotion_pipeline:
        print(f"\nRunning Transformer analyses (Sentiment & Emotion using
'{TEXT_COL}')...")
        num_rows = len(df)
        # Initialize lists for the 4 transformer outputs
        transformer_sent_labels = [None] * num_rows; transformer_sent_scores =
[None] * num_rows
        transformer_emot_top3_str = [None] * num_rows # For JSON string
        transformer_emot_dominant = [None] * num_rows # For dominant label

        # Process in batches
        for i in range(0, num_rows, BATCH_SIZE):
            batch_texts = df[TEXT_COL].iloc[i:min(i + BATCH_SIZE,
num_rows)].tolist()
            # Get the 4 results from the batch function
            (s_labels, s_scores,
             e_top3_str, e_dominant) = get_transformer_analyses_batch(batch_texts)

            # Assign results
            start_idx, end_idx = i, min(i + BATCH_SIZE, num_rows)
            transformer_sent_labels[start_idx:end_idx] = s_labels
            transformer_sent_scores[start_idx:end_idx] = s_scores
            transformer_emot_top3_str[start_idx:end_idx] = e_top3_str # Assign JSON
string list
            transformer_emot_dominant[start_idx:end_idx] = e_dominant   # Assign
dominant label list

            # Progress printing
            if (i // BATCH_SIZE + 1) % 10 == 0:
                 processed_count = min(i + BATCH_SIZE, num_rows)
                 total_batches = (num_rows + BATCH_SIZE - 1) // BATCH_SIZE
                 print(f"  Transformer Batch {i // BATCH_SIZE + 1} / {total_batches}
({processed_count}/{num_rows} rows)")

        # Assign lists to DataFrame columns
```

```
        df[NEW_COL_TRANSFORMER_SENT_LABEL] = transformer_sent_labels
        df[NEW_COL_TRANSFORMER_SENT_SCORE] = transformer_sent_scores
        df[NEW_COL_HF_EMOTION_TOP3_STR] = transformer_emot_top3_str # New column
        df[NEW_COL_HF_EMOTION_DOMINANT] = transformer_emot_dominant     # New column
        print("Transformer analyses complete.")
    else:
        print("\nSkipping Transformer analyses as models did not load.")
        df[NEW_COL_TRANSFORMER_SENT_LABEL] = "N/A";
df[NEW_COL_TRANSFORMER_SENT_SCORE] = None
        df[NEW_COL_HF_EMOTION_TOP3_STR] = "N/A"; df[NEW_COL_HF_EMOTION_DOMINANT] =
"N/A"


    # --- Insert new columns (Step 4) ---
    print(f"\nInserting new columns after '{LAST_EXISTING_COL}' (or at end)...")
    # Update the list of new columns in desired order (10 analysis + 2 cleaning =
12)
    new_cols_ordered = [
        NEW_COL_CLEANED_V2, NEW_COL_CLEANED_V2_WC,
        NEW_COL_VADER_SCORE, NEW_COL_VADER_LABEL,
        NEW_COL_T2E_SCORES, NEW_COL_T2E_LABEL,
        NEW_COL_TRANSFORMER_SENT_LABEL, NEW_COL_TRANSFORMER_SENT_SCORE,
        NEW_COL_HF_EMOTION_TOP3_STR, # HF Emotion Top 3 scores (JSON)
        NEW_COL_HF_EMOTION_DOMINANT,  # HF Emotion Dominant Label
    ]
    # ... (Rest of insertion logic - same as before, just uses the new list) ...
    existing_new_cols = [col for col in new_cols_ordered if col in df.columns]
    if existing_new_cols:
        df_new_cols = df[existing_new_cols].copy()
        df = df.drop(columns=existing_new_cols)
        for i, col_name in enumerate(new_cols_ordered):
            if col_name in df_new_cols.columns: df.insert(insert_loc + i, col_name,
df_new_cols[col_name])
            else: df.insert(insert_loc + i, col_name, "N/A")
    else: print("No new columns were generated to insert.")


    # --- Save Results (Step 5) ---
    # ... (Same save logic as before) ...
    print(f"\nSaving results to {OUTPUT_FILE}...")
    try:
        if NEW_COL_T2E_SCORES in df.columns: df[NEW_COL_T2E_SCORES] =
df[NEW_COL_T2E_SCORES].astype(str)
        # No explicit conversion needed for HF_EMOTION_TOP3_STR as it's already a
JSON string
        df.to_excel(OUTPUT_FILE, index=False, engine="openpyxl")
        print("Successfully saved updated data.")
    except Exception as e: print(f"Error saving Excel file: {e}")

    end_time = time.time()
```

```
    print(f"\nScript finished in {end_time - start_time:.2f} seconds.")
    print("Reminder: Interpret AI results with caution.")

# --- Run the main function ---
if __name__ == "__main__":
    main()

    # ---------------------------------------------------------------------------------------
    # 3. TOPIC MODELING (LDA & LSA) - Saved to New Sheets
    # ---------------------------------------------------------------------------------------
    print("🟢 Running Topic Modeling...")

    vectorizer = TfidfVectorizer(max_df=0.95, min_df=2, stop_words='english')
    tfidf = vectorizer.fit_transform(df["Original Text"].fillna(""))

    def extract_topics(model, name):
        print(f"🟢 Extracting topics using {name}...")
        model.fit(tfidf)
        if not hasattr(model, "components_") or model.components_.shape[0] == 0:
            print(f"❌ Error: No topics extracted for {name}. Check input data.")
            return pd.DataFrame()
        topic_dict = {
            f"Topic {i+1}": ", ".join([vectorizer.get_feature_names_out()[idx] for
idx in topic.argsort()[:-11:-1]])
            for i, topic in enumerate(model.components_)
        }
        if not topic_dict:
            print(f"❌ Error: Topic dictionary is empty for {name}.")
            return pd.DataFrame(columns=[f"Topic {i+1}" for i in
range(model.n_components)])
        return pd.DataFrame(topic_dict, index=[0])

    lda_results_df = extract_topics(LatentDirichletAllocation(n_components=5,
random_state=42), "LDA")
    lsa_results_df = extract_topics(TruncatedSVD(n_components=5, random_state=42),
"LSA")
    print("✅ Topic Modeling completed.")

    # ---------------------------------------------------------------------------------------
    # 4. UPDATE "Scraped Data" SHEET WITHOUT ALTERING EXISTING FORMATTING
    # ---------------------------------------------------------------------------------------
    print("🟢 Updating 'Scraped Data' sheet with new analysis columns without
altering existing formatting...")
    wb = load_workbook(file_path)
    ws = wb["Scraped Data"]

    # Find the next available column (preserve all existing formatting)
    last_col = ws.max_column
    new_columns = ["Sentiment Compound Score", "Sentiment Label", "Emotion Scores",
"Emotion Label"]
```

```
    # Write headers into the first row (starting at the next empty column)
    for i, header in enumerate(new_columns, start=last_col + 1):
        ws.cell(row=1, column=i, value=header)

    # Write new data rows (data starts on row 2)
    for idx, row in df.iterrows():
        excel_row = idx + 2  # because row 1 is header
        ws.cell(row=excel_row, column=last_col + 1, value=row["Sentiment Compound
Score"])
        ws.cell(row=excel_row, column=last_col + 2, value=row["Sentiment Label"])
        # Save the Emotion Scores as string (so the dictionary appears properly)
        ws.cell(row=excel_row, column=last_col + 3, value=str(row["Emotion
Scores"]))
        ws.cell(row=excel_row, column=last_col + 4, value=row["Emotion Label"])

    wb.save(file_path)
    print("✅ 'Scraped Data' sheet updated successfully while preserving existing
formatting.")

    # ------------------------------------------------------------------------------
    # 5. Save Topic Modeling Results to New Sheets
    # ------------------------------------------------------------------------------
    print("🔄 Saving topic modeling results to Excel...")
    wb = load_workbook(file_path)
    sheet_names = wb.sheetnames

    # Remove existing topic modeling sheets if they exist
    for sheet in ["LDA Results", "LSA Results"]:
        if sheet in sheet_names:
            del wb[sheet]
    wb.save(file_path)

    # Append new sheets using Pandas (formatting is not an issue for these new
sheets)
    with pd.ExcelWriter(file_path, engine='openpyxl', mode='a') as writer:
        lda_results_df.to_excel(writer, sheet_name="LDA Results", index=False)
        lsa_results_df.to_excel(writer, sheet_name="LSA Results", index=False)
    print("✅ Topic modeling results saved successfully.")

    # ------------------------------------------------------------------------------
    # 6. Delete Old Visualizations
    # ------------------------------------------------------------------------------
    print("🔄 Deleting old visualizations...")
    visualization_files = ['Sentiment_Distribution.png', 'Common_Emotions.png',
'Sentiment_Trend_Over_Time.png']
    for file in visualization_files:
        file_path_png = os.path.join(figures_dir, file)
        if os.path.exists(file_path_png):
            os.remove(file_path_png)
```

```
        print(f"🗑️ Deleted: {file_path_png}")
    print("✅ Old visualizations deleted.")

    # ------------------------------------------------------------------------------
    # 7. Generate New Visualizations
    # ------------------------------------------------------------------------------
    print("🎨 Generating new visualizations...")

    # Visualization 1: Sentiment Distribution (using the Sentiment Label)
    plt.figure(figsize=(8, 6))
    df['Sentiment Label'].value_counts().plot(kind='bar', title='Sentiment
Distribution')
    plt.savefig(os.path.join(figures_dir, 'Sentiment_Distribution.png'))
    plt.close()
    print("📊 Sentiment Distribution saved.")

    # Visualization 2: Most Common Emotions (using the Emotion Label)
    plt.figure(figsize=(10, 6))
    common_emotions = df['Emotion Label'].value_counts().head(10)
    common_emotions.plot(kind='bar', title='Most Common Emotions')
    plt.savefig(os.path.join(figures_dir, 'Common_Emotions.png'))
    plt.close()
    print("📊 Common Emotions saved.")

    # Visualization 3: Sentiment Trend Over Time (if a "Date" column exists)
    if "Date" in df.columns:
        df['Date'] = pd.to_datetime(df['Date'], errors='coerce')
        df.set_index('Date', inplace=True)
        sentiment_trends = df.resample('M')["Sentiment Compound Score"].mean()
        plt.figure(figsize=(12, 6))
        sentiment_trends.plot(kind='line', title='Sentiment Trend Over Time')
        plt.savefig(os.path.join(figures_dir, 'Sentiment_Trend_Over_Time.png'))
        plt.close()
        print("📊 Sentiment Trend Over Time saved.")

    print("✅ Visualizations completed successfully.")
```
