# Supplementary material for: Human Papillomavirus Vaccine Discourse and Sentiment on Reddit Before and After COVID-19: Mixed Methods Retrospective Cross-Sectional Study
Source: J Med Internet Res. 2026 May 19;28:e83558. doi: 10.2196/83558 (PMC13186556; doi:10.2196/83558)
Supplement: Multimedia Appendix 3 [file jmir-v28-e83558-s003.pdf]

```
# Libraries
import praw
import pandas as pd
from datetime import datetime
import time
import os
import re
import nltk
from openpyxl import load_workbook
from openpyxl.styles import numbers, Alignment, Font

#---------------------------------------------------------------------------------
---------------------#

# Project-specific elements
PROJECT_NAME = "HPV Vaccine"

# Define common keyword groups
HPV_SUBREDDIT_KEYWORDS = [
    'vaccine', 'vaccines', 'vaccinated', 'vaccination', 'vaccinations', 'vax',
'vaxxed', 'unvaxxed',
    'gardasil', 'cervarix', 'anti-vax', 'antivax', 'antivaccine', 'anti-vaccine',
'pro-vax',
    'jab', 'shot', 'immunization', 'immunized'
]

VACCINE_RELATED__VACCINE_SUBREDDIT_KEYWORDS = [
    'HPV', 'HPV vaccine', 'HPV shot', 'gardasil', 'cervarix', 'human papillomavirus'
]

# Mapping keywords to specific subreddits
KEYWORDS_MAPPING = {
    'HPV': HPV_SUBREDDIT_KEYWORDS,
}

#Keyword logic to be used for all other subreddits
DEFAULT_KEYWORDS = [
    'hpv vaccine', 'hpv shot', 'gardasil', 'cervarix'
]

# Define the HPV-related and Vaccine-related word pools
HPV_TERMS = [
    'hpv', 'human papillomavirus', 'papillomavirus', 'genital warts',
    'cervix cancer', 'cervical cancer', 'oropharyngeal cancer', 'throat cancer',
    'anal cancer', 'rectal cancer', 'vagine cancer', 'vaginal cancer', 'vulva
cancer',
    'vulvar cancer', 'penis cancer', 'penile cancer', 'head and neck cancer',
    'oropharynx cancer', 'mouth cancer', 'tonsil cancer', 'tongue cancer',
]
```

```
VACCINE_TERMS = [
    'vaccine', 'vaccines', 'vaccinated', 'vaccination', 'vaccinations', 'vax',
    'vaxxed', 'unvaxxed', 'gardasil', 'cervarix', 'anti-vax', 'antivax',
    'antivaccine', 'anti-vaccine', 'pro-vax', 'jab', 'shot', 'immunization',
'immunized'
]

SUBREDDIT_LIST = [
    'HPV', 'VACCINES', 'vaxxhappened', 'Vaccine', 'TheVaccines', 'ForkedVaccines',
'DebaseVaccines', 'VaccinesMeds', 'MomsAgainstVaccines',
    'StopMandatoryVaccines', 'antivax', 'vaccineautismevidence', 'vaccineskeptics',
'unvaccinated', 'VaccineStrike', 'DebateVaccines',
    'Xennials', 'AskDocs', 'Health', 'MensRights', 'medicine', 'TwoXChromosomes',
'AskWomenOver30', 'PeterAttia', 'TheGirlSurvivalGuide',
    'pharmacy', 'NoStupidQuestions', 'askgaybros', 'Warts', 'CervicalCancer',
'AskGaybrosOver30', 'Advice', 'WomenOver40', 'PreCervicalCancer',
    'Frugal', 'AMA', 'r/pediatrics', 'NewParents', 'beyondthebump',
'ScienceBasedParenting', 'autism', 'news', 'AskConservatives', 'skeptic',
    'Autism_Parenting', 'conspiracy', 'HealthInsurance', 'relationship_advice',
'HealthAnxiety', 'HeadandNeckCancer', 'HNSCC','AskDentists','cancer',
'otolaryngology'
]

# Lists the output file directory file
OUTPUT_DIR = f'/Users/Sean/Desktop/Python/Reddit ENT
Projects/Active/{PROJECT_NAME}/Data Files'
os.makedirs(OUTPUT_DIR, exist_ok=True)
OUTPUT_FILE_EXCEL =
f"{OUTPUT_DIR}/{PROJECT_NAME}_Scraped_Data_more_subreddits_Less_Strict.xlsx"

# General settings
LIMIT = None  # Set a limit for posts/comments to scrape, None for no limit
POST_KEYWORD_REQUIRED = lambda subreddit: True
COMMENT_KEYWORD_REQUIRED = lambda subreddit: True

#-----------------------------------------------------------------------------------
---------------------#

# Ensure stopwords are downloaded
try:
    nltk.data.find('corpora/stopwords.zip')
except LookupError:
    nltk.download('stopwords')
stop_words = set(nltk.corpus.stopwords.words('english'))

# Set up Reddit app credentials
reddit = praw.Reddit(
    client_id='uW7mU0k2SaWnkLn3kbGxDQ',
    client_secret='PuCbZFuWc7mYZJko86W-JaegiZLeMQ',
    user_agent='MyRedditScraper/0.1 by seanatl2019',
```

```
    check_for_async=False
)

def get_keywords(subreddit):
    return KEYWORDS_MAPPING.get(subreddit, DEFAULT_KEYWORDS)

def find_inclusion_keywords(text, subreddit):
    if isinstance(text, str):
        text_lower = text.lower()

        # Step 1: Check if the subreddit has explicit keyword mappings
        keywords = get_keywords(subreddit)

        # Sort keywords by length (longer keywords are prioritized for better
matching)
        keywords = sorted(keywords, key=len, reverse=True)

        # Step 1a: Check if any predefined keywords are in the text
        keywords_found = []
        for keyword in keywords:
            if re.search(re.escape(keyword), text_lower):
                # Add the keyword if not already matched to avoid duplicates
                if keyword not in keywords_found:
                    keywords_found.append(keyword)

        # Step 1b: If any predefined keywords are found, return them
        if keywords_found:
            return ", ".join(keywords_found)

        # Step 2: For subreddits **without explicit mappings**, apply backup keyword
matching
        if subreddit not in KEYWORDS_MAPPING:

            # Step 2a: Look for at least one term from the HPV-related word pool
            found_hpv_terms = [kw for kw in HPV_TERMS if
re.search(rf"\b{re.escape(kw)}\b", text_lower)]

            # Step 2b: Look for at least one term from the Vaccine-related word pool
            found_vaccine_terms = [kw for kw in VACCINE_TERMS if
re.search(rf"\b{re.escape(kw)}\b", text_lower)]

            # Step 2c: If both an HPV term AND a vaccine term are found, return all
matched words
            if found_hpv_terms and found_vaccine_terms:
                return ", ".join(found_hpv_terms + found_vaccine_terms)

    # Step 3: If no matches are found, return None
    return None

def preprocess_text(text):
```

```
    try:
        text = text.lower()
        text = re.sub(r'http\S+|www\S+|https\S+', '', text, flags=re.MULTILINE)
        emoji_pattern = re.compile(
            "["  # Start of character set
            u"\\U0001F600-\\U0001F64F"  # Emoticons
            u"\\U0001F300-\\U0001F5FF"  # Misc Symbols and Pictographs
            u"\\U0001F680-\\U0001F6FF"  # Transport and Map Symbols
            u"\\U0001F700-\\U0001F77F"  # Alchemical Symbols
            u"\\U0001F780-\\U0001F7FF"  # Geometric Shapes Extended
            u"\\U0001F800-\\U0001F8FF"  # Supplemental Arrows-C
            u"\\U0001F900-\\U0001F9FF"  # Supplemental Symbols and Pictographs
            "]+", flags=re.UNICODE
        )

        text = emoji_pattern.sub(r'', text)  # Remove emojis
        text = re.sub(r'[^\w\s]', '', text)  # Remove non-alphanumeric characters
except spaces
        text = re.sub(r'\d+', '', text)  # Remove numbers
        tokens = [word for word in text.split() if word not in stop_words]  #
Tokenize and remove stopwords
        return ' '.join(tokens)
    except Exception as e:
        print(f"Error during text preprocessing: {str(e)}")
        return text

def incremental_save(data, file_path):
    temp_file = file_path.replace('.xlsx', '_temp.xlsx')
    try:
        with pd.ExcelWriter(temp_file, engine='openpyxl') as writer:
            data.to_excel(writer, index=False)
        os.replace(temp_file, file_path)
    except Exception as e:
        print(f"Error saving file incrementally: {e}")

def get_comments(comment_forest, submission, subreddit_name, keyword_required):
    comments_data = []
    try:
        for comment in comment_forest:
            if isinstance(comment, praw.models.Comment):
                keywords_used = None if not keyword_required else
find_inclusion_keywords(comment.body, subreddit_name)
                if not keyword_required or keywords_used:
                    comment_data = {
                        'Subreddit': subreddit_name,
                        'Original Text': comment.body,
                        'Flair': comment.author_flair_text,
                        'Date': datetime.fromtimestamp(comment.created_utc),
                        'Upvotes': comment.score,
                        'ID': comment.id,
```

```
                        'Parent ID': comment.parent_id,
                        'Author': comment.author.name if comment.author else 'N/A',
                        'Type': 'Comment',
                        'Post ID': submission.id,
                        'Keywords': keywords_used,
                    }
                    comments_data.append(comment_data)
    except Exception as e:
        print(f"Error processing comments: {e}")
    return comments_data

def get_posts_and_comments(subreddit_name, post_keyword_required,
comment_keyword_required, limit=None):
    subreddit = reddit.subreddit(subreddit_name)
    posts_and_comments_data = pd.DataFrame()
    post_counter = 0
    comment_counter = 0
    date_range = {'start_date': None, 'end_date': None}  # Track date range
dynamically

    endpoints = {
        'new': subreddit.new(limit=limit),
        'top': subreddit.top(limit=limit),
        'hot': subreddit.hot(limit=limit)
    }

    retry_attempts = 3
    backoff_time = 60

    for attempt in range(retry_attempts):
        try:
            for endpoint_name, submissions in endpoints.items():
                for submission in submissions:
                    combined_text = f"{submission.title}
{submission.selftext}".strip()
                    keywords_used = None if not post_keyword_required else
find_inclusion_keywords(combined_text, subreddit_name)
                    if not post_keyword_required or keywords_used:
                        post_date = datetime.fromtimestamp(submission.created_utc)

                        # Update dynamic date range
                        if not date_range['start_date'] or post_date <
date_range['start_date']:
                            date_range['start_date'] = post_date
                        if not date_range['end_date'] or post_date >
date_range['end_date']:
                            date_range['end_date'] = post_date

                        submission_data = pd.DataFrame([{
                            'Subreddit': subreddit_name,
```

```
                            'Original Text': combined_text,
                            'Flair': submission.link_flair_text,
                            'Date': post_date,
                            'Upvotes': submission.score,
                            'ID': submission.id,
                            'Parent ID': None,
                            'Author': submission.author.name if submission.author
else 'N/A',
                            'Type': 'Post',
                            'Post ID': submission.id,
                            'Keywords': keywords_used,
                        }])
                        posts_and_comments_data =
pd.concat([posts_and_comments_data, submission_data], ignore_index=True)

                        # Process comments
                        submission.comments.replace_more(limit=None)
                        comment_data = get_comments(submission.comments.list(),
submission, subreddit_name, comment_keyword_required)
                        comments_df = pd.DataFrame(comment_data)
                        posts_and_comments_data =
pd.concat([posts_and_comments_data, comments_df], ignore_index=True)

                        # Drop duplicates to maintain unique posts and comments
                        posts_and_comments_data.drop_duplicates(subset='ID',
inplace=True)

                        # Update unique counters
                        post_counter =
posts_and_comments_data[posts_and_comments_data['Type'] == 'Post']['ID'].nunique()
                        comment_counter =
posts_and_comments_data[posts_and_comments_data['Type'] ==
'Comment']['ID'].nunique()

                        # Print progress for every 50 unique posts
                        if post_counter % 50 == 0:
                            print(f"Subreddit: {subreddit_name} | Unique Posts:
{post_counter} | Unique Comments: {comment_counter} | Date Range:
{date_range['start_date'].strftime('%b %d, %Y')} -
{date_range['end_date'].strftime('%b %d, %Y')}")

            print(f"Final counts for subreddit {subreddit_name}: Unique Posts:
{post_counter} | Unique Comments: {comment_counter}")
            break
        except praw.exceptions.RedditAPIException as e:
            if 'RATELIMIT' in str(e):
                print(f"Rate limit hit, sleeping for {backoff_time} seconds.")
                time.sleep(backoff_time)
                backoff_time *= 2
            else:
```

```
                print(f"APIException occurred: {e}")
        except Exception as e:
            print(f"An unexpected error occurred during attempt {attempt + 1}: {e}")
            time.sleep(backoff_time)
    return posts_and_comments_data, post_counter, comment_counter

# Retrieve the number of members in a subreddit
def get_subreddit_member_count(subreddit_name):
    try:
        subreddit = reddit.subreddit(subreddit_name)
        return subreddit.subscribers
    except Exception as e:
        print(f"Error fetching member count for subreddit {subreddit_name}: {e}")
        return None

# Start of main execution
os.makedirs(OUTPUT_DIR, exist_ok=True)

# Initialize a DataFrame to store all posts and comments
all_posts_and_comments = pd.DataFrame()
summary = []

# Loop through each subreddit to scrape data
for subreddit in SUBREDDIT_LIST:
    try:
        print(f"Scraping subreddit: {subreddit}...")
        posts_comments, post_counter, comment_counter = get_posts_and_comments(
            subreddit, POST_KEYWORD_REQUIRED(subreddit),
COMMENT_KEYWORD_REQUIRED(subreddit), limit=LIMIT
        )
        all_posts_and_comments = pd.concat([all_posts_and_comments, posts_comments],
ignore_index=True)

        # Remove duplicates after appending
        all_posts_and_comments.drop_duplicates(subset='ID', inplace=True)

        # Incremental saving after every subreddit is processed
        all_posts_and_comments = all_posts_and_comments.sort_values(by='Date',
ascending=False)
        incremental_save(all_posts_and_comments, OUTPUT_FILE_EXCEL)

    except Exception as e:
        print(f"Failed to get posts for subreddit {subreddit}: {e}")
        time.sleep(30)

# If we have any data at all, do processing
if not all_posts_and_comments.empty:
    # 1.) Scraped Data: write + format
    print("Generating Scraped Data Sheet...")
```

```
    # Pre-process columns
    all_posts_and_comments['Pre-Processed Text'] = all_posts_and_comments['Original
Text'].apply(preprocess_text)
    all_posts_and_comments['Original Word Count'] = all_posts_and_comments['Original
Text'].str.split().str.len()
    all_posts_and_comments['Pre-Processed Word Count'] =
all_posts_and_comments['Pre-Processed Text'].str.split().str.len()

    # Reorder columns
    column_order = [
        'Subreddit', 'Original Text', 'Original Word Count', 'Pre-Processed Text',
        'Pre-Processed Word Count', 'Flair', 'Date', 'Type', 'Keywords', 'Upvotes',
        'Author', 'ID', 'Parent ID', 'Post ID'
    ]
    all_posts_and_comments = all_posts_and_comments[column_order]

    # Write "Scraped Data" (fresh, overwriting if it already exists)
    all_posts_and_comments.to_excel(OUTPUT_FILE_EXCEL, index=False,
sheet_name='Scraped Data')

    # Now open workbook and format "Scraped Data"
    workbook = load_workbook(OUTPUT_FILE_EXCEL)
    scraped_data_sheet = workbook["Scraped Data"]

    # Define widths
    column_width_map = {
        "Subreddit": 10.83,
        "Flair": 10.83,
        "Original Word Count": 16.83,
        "Pre-Processed Word Count": 22.83,
        "Date": 18.83,
        "Type": 8.83,
        "Upvotes": 7.83,
        "Author": 18.83,
        "ID": 8.83,
        "Parent ID": 11.83,
        "Post ID": 8.83,
        "Original Text": 30.83,
        "Pre-Processed Text": 30.83,
        "Keywords": 15.83
    }

    header = [cell.value for cell in scraped_data_sheet[1]]
    for column_name, desired_width in column_width_map.items():
        if column_name in header:
            col_idx = header.index(column_name) + 1
            col_letter = scraped_data_sheet.cell(row=1,
column=col_idx).column_letter
            scraped_data_sheet.column_dimensions[col_letter].width = desired_width
```

```
    # Center-align some columns
    columns_to_center = [
        "Original Word Count", "Pre-Processed Word Count", "Date",
        "Type", "Upvotes", "Author", "ID", "Parent ID", "Post ID"
    ]
    center_col_indices = [header.index(c) + 1 for c in columns_to_center if c in
header]
    for col_idx in center_col_indices:
        for cell_range in scraped_data_sheet.iter_cols(
            min_col=col_idx, max_col=col_idx,
            min_row=2, max_row=scraped_data_sheet.max_row
        ):
            for cell in cell_range:
                cell.alignment = Alignment(horizontal="center", vertical="center")

    # Save changes to workbook
    workbook.save(OUTPUT_FILE_EXCEL)

    print("Scraped Data sheet added successfully!")

    # 2.) Summary: write + format
    # Build summary data
    summary = []
    for subreddit in SUBREDDIT_LIST:
        sub_data = all_posts_and_comments[all_posts_and_comments['Subreddit'] ==
subreddit]
        if not sub_data.empty:
            member_count = get_subreddit_member_count(subreddit)
            total_posts = len(sub_data[sub_data['Type'] == 'Post'])
            total_comments = len(sub_data[sub_data['Type'] == 'Comment'])
            total_posts_comments = total_posts + total_comments
            unique_post_authors = sub_data[sub_data['Type'] ==
'Post']['Author'].nunique()
            unique_comment_authors = sub_data[sub_data['Type'] ==
'Comment']['Author'].nunique()
            unique_users = sub_data['Author'].nunique()
            avg_comments_per_post = total_comments / total_posts if total_posts else
0
            avg_posts_comments_per_user = len(sub_data) / unique_users if
unique_users else 0
            avg_words_per_post = sub_data[sub_data['Type'] == 'Post']['Original Word
Count'].mean()
            avg_words_per_comment = sub_data[sub_data['Type'] ==
'Comment']['Original Word Count'].mean()

            summary.append({
                'Subreddit': subreddit,
                'Unique Posts': total_posts,
                'Unique Comments': total_comments,
                'Total Posts/Comments': total_posts_comments,
```

```
                'Unique Post Authors': unique_post_authors,
                'Unique Comment Authors': unique_comment_authors,
                'Total Unique Users': unique_users,
                'Member Count': member_count,
                'Date Range Start': sub_data['Date'].min().date(),
                'Date Range End': sub_data['Date'].max().date(),
                'Avg Comments Per Post': avg_comments_per_post,
                'Avg Posts/Comments Per User': avg_posts_comments_per_user,
                'Avg Words Per Post': avg_words_per_post,
                'Avg Words Per Comment': avg_words_per_comment
            })

    summary_df = pd.DataFrame(summary)

    # Totals row
    totals_row = {
        'Subreddit': 'Totals/Averages',
        'Unique Posts': all_posts_and_comments[all_posts_and_comments['Type'] ==
'Post']
                       .drop_duplicates(subset='ID')['ID'].nunique(),
        'Unique Comments': all_posts_and_comments[all_posts_and_comments['Type'] ==
'Comment']
                          .drop_duplicates(subset='ID')['ID'].nunique(),
        'Total Posts/Comments':
all_posts_and_comments.drop_duplicates(subset='ID').shape[0],
        'Unique Post Authors': summary_df['Unique Post Authors'].sum(),
        'Unique Comment Authors': summary_df['Unique Comment Authors'].sum(),
        'Total Unique Users': summary_df['Total Unique Users'].sum(),
        'Member Count': summary_df['Member Count'].sum(),
        'Date Range Start': all_posts_and_comments['Date'].min().date(),
        'Date Range End': all_posts_and_comments['Date'].max().date(),
        'Avg Comments Per Post': summary_df['Avg Comments Per Post'].mean(),
        'Avg Posts/Comments Per User': summary_df['Avg Posts/Comments Per
User'].mean(),
        'Avg Words Per Post': summary_df['Avg Words Per Post'].mean(),
        'Avg Words Per Comment': summary_df['Avg Words Per Comment'].mean()
    }
    summary_df = pd.concat([summary_df, pd.DataFrame([totals_row])],
ignore_index=True)

    # Write "Summary" (append mode)
    with pd.ExcelWriter(OUTPUT_FILE_EXCEL, engine='openpyxl', mode='a',
if_sheet_exists='replace') as writer:
        summary_df.to_excel(writer, sheet_name='Summary', index=False)

    # Now format "Summary"
    workbook = load_workbook(OUTPUT_FILE_EXCEL)
    summary_sheet = workbook["Summary"]

    # Zero-decimal columns
```

```
    columns_zero_decimal = [
        "Unique Posts", "Unique Comments", "Total Posts/Comments",
        "Unique Post Authors", "Unique Comment Authors",
        "Total Unique Users", "Member Count"
    ]
    # Two-decimal columns
    columns_two_decimal = [
        "Avg Comments Per Post", "Avg Posts/Comments Per User",
        "Avg Words Per Post", "Avg Words Per Comment"
    ]

    header = [cell.value for cell in summary_sheet[1]]
    zero_decimal_indices = [header.index(c) + 1 for c in columns_zero_decimal if c
in header]
    two_decimal_indices = [header.index(c) + 1 for c in columns_two_decimal if c in
header]

    # Format numeric columns and center align everything
    for row in summary_sheet.iter_rows(min_row=2, max_row=summary_sheet.max_row):
        for cell in row:
            # If it's in zero-decimal columns
            if cell.column in zero_decimal_indices and isinstance(cell.value, (int,
float)):
                cell.number_format = '#,##0'
            # If it's in two-decimal columns
            elif cell.column in two_decimal_indices and isinstance(cell.value, (int,
float)):
                cell.number_format = '#,##0.00'
            # Center align
            cell.alignment = Alignment(horizontal="center", vertical="center")

    # Bold the "Totals/Averages" row
    for row in summary_sheet.iter_rows(min_row=2, max_row=summary_sheet.max_row):
        if row[0].value == "Totals/Averages":
            for cell in row:
                cell.font = Font(bold=True)
            break

    # Summary sheet column widths
    summary_column_width_map = {
        "Subreddit": 13,
        "Unique Posts": 11,
        "Unique Comments": 15,
        "Total Posts/Comments": 18,
        "Unique Post Authors": 17,
        "Unique Comment Authors": 21,
        "Total Unique Users": 15,
        "Member Count": 13,
        "Date Range Start": 13,
        "Date Range End": 13,
```

```
        "Avg Comments Per Post": 19,
        "Avg Posts/Comments Per User": 24,
        "Avg Words Per Post": 16,
        "Avg Words Per Comment": 20
    }
    for col_name, width_val in summary_column_width_map.items():
        if col_name in header:
            idx = header.index(col_name) + 1
            letter = summary_sheet.cell(row=1, column=idx).column_letter
            summary_sheet.column_dimensions[letter].width = width_val

    # Save changes to workbook
    workbook.save(OUTPUT_FILE_EXCEL)

    print("Summary sheet added successfully!")

    # 3.) Keyword Breakdown: write + format
    # Flatten the list of all keywords from the "Keywords" column
    keyword_list = all_posts_and_comments['Keywords'].dropna().str.split(',
').explode()

    # Count occurrences of each keyword
    keyword_counts = keyword_list.value_counts().reset_index()
    keyword_counts.columns = ['Keyword', 'Count']

    # Write "Keyword Breakdown" sheet
    with pd.ExcelWriter(OUTPUT_FILE_EXCEL, engine='openpyxl', mode='a',
if_sheet_exists='replace') as writer:
        keyword_counts.to_excel(writer, sheet_name='Keyword Breakdown', index=False)

    # Now format "Keyword Breakdown"
    workbook = load_workbook(OUTPUT_FILE_EXCEL)
    keyword_sheet = workbook["Keyword Breakdown"]

    # Center-align all columns
    for col in keyword_sheet.iter_cols(min_col=1, max_col=2, min_row=2,
max_row=keyword_sheet.max_row):
        for cell in col:
            cell.alignment = Alignment(horizontal="center", vertical="center")

    # Set column widths
    keyword_sheet.column_dimensions["A"].width = 25  # "Keyword" column
    keyword_sheet.column_dimensions["B"].width = 12  # "Count" column

    # Save changes to workbook
    workbook.save(OUTPUT_FILE_EXCEL)

    print("Keyword Breakdown sheet added successfully!")

    # 4.) Yearly Sheets: write + format
```

```
    # Build "Yearly Totals"
    yearly_totals = (
        all_posts_and_comments
        .groupby(all_posts_and_comments['Date'].dt.year)
        .size()
        .reset_index(name='Total Posts/Comments')
        .rename(columns={'Date': 'Year'})
    )

    # Write "Yearly Totals" and each "Subreddit_Yearly"
    with pd.ExcelWriter(OUTPUT_FILE_EXCEL, engine='openpyxl', mode='a',
if_sheet_exists='replace') as writer:
        yearly_totals.to_excel(writer, sheet_name='Yearly Totals', index=False)
        for sr in SUBREDDIT_LIST:
            sub_data = all_posts_and_comments[all_posts_and_comments['Subreddit'] ==
sr]
            if not sub_data.empty:
                yearly_data = (
                    sub_data
                    .groupby(sub_data['Date'].dt.year)
                    .size()
                    .reset_index(name='Total Posts/Comments')
                    .rename(columns={'Date': 'Year'})
                )
                yearly_sheet_name = f"{sr}_Yearly"
                yearly_data.to_excel(writer, sheet_name=yearly_sheet_name,
index=False)

    # Now format the newly written Yearly sheets
    workbook = load_workbook(OUTPUT_FILE_EXCEL)

    # Format "Yearly Totals"
    if "Yearly Totals" in workbook.sheetnames:
        y_sheet = workbook["Yearly Totals"]
        # Grab header
        header = [cell.value for cell in y_sheet[1]]
        if "Year" in header and "Total Posts/Comments" in header:
            year_idx = header.index("Year") + 1
            total_idx = header.index("Total Posts/Comments") + 1

            # Center alignment
            for row in y_sheet.iter_rows(min_row=2, max_row=y_sheet.max_row):
                row[year_idx - 1].alignment = Alignment(horizontal="center",
vertical="center")
                row[total_idx - 1].alignment = Alignment(horizontal="center",
vertical="center")

            # Fixed widths
            year_letter = y_sheet.cell(row=1, column=year_idx).column_letter
            total_letter = y_sheet.cell(row=1, column=total_idx).column_letter
```

```
            y_sheet.column_dimensions[year_letter].width = 10
            y_sheet.column_dimensions[total_letter].width = 25

    # Format each "xxx_Yearly"
    for sheet_name in workbook.sheetnames:
        if sheet_name.endswith("_Yearly"):
            sub_yr_sheet = workbook[sheet_name]
            header = [cell.value for cell in sub_yr_sheet[1]]
            if "Year" in header and "Total Posts/Comments" in header:
                year_idx = header.index("Year") + 1
                total_idx = header.index("Total Posts/Comments") + 1
                # Center align
                for row in sub_yr_sheet.iter_rows(min_row=2,
max_row=sub_yr_sheet.max_row):
                    row[year_idx - 1].alignment = Alignment(horizontal="center",
vertical="center")
                    row[total_idx - 1].alignment = Alignment(horizontal="center",
vertical="center")
                # Column widths
                yr_letter = sub_yr_sheet.cell(row=1, column=year_idx).column_letter
                tot_letter = sub_yr_sheet.cell(row=1,
column=total_idx).column_letter
                sub_yr_sheet.column_dimensions[yr_letter].width = 10
                sub_yr_sheet.column_dimensions[tot_letter].width = 25

    # Save changes after formatting all sheets
    workbook.save(OUTPUT_FILE_EXCEL)

    print("Yearly Totals sheet added successfully!")

else:
    # If no data
    print("No data was scraped; skipping processing and output generation.")

# Print final status
print("Scraping complete.")
print(f"Total unique posts scraped: {totals_row['Unique Posts']}")
print(f"Total unique comments scraped: {totals_row['Unique Comments']}")
print(f"Date range: {totals_row['Date Range Start']} to {totals_row['Date Range
End']}")
print("Script execution complete.")
```
